# Relevance of 3D Cholangiography and Transient Elastography to Assess Cystic Fibrosis-Associated Liver Disease?

**DOI:** 10.1155/2016/4592702

**Published:** 2016-06-26

**Authors:** C. Lemaitre, S. Dominique, E. Billoud, M. Eliezer, H. Montialoux, M. Quillard, G. Riachi, E. Koning, H. Morisse-Pradier, G. Savoye, C. Savoye-Collet, O. Goria

**Affiliations:** ^1^Department of Gastroenterology and Hepatology, Rouen University Hospital, Rouen, France; ^2^Department of Pneumology, Adult Cystic Fibrosis Centre, Rouen University Hospital, Rouen, France; ^3^Department of Gastroenterology, Le Havre General Hospital, Le Havre, France; ^4^Department of Radiology, Rouen University Hospital, Rouen, France; ^5^Clinical Biology Institute, Rouen University Hospital, Rouen, France; ^6^INSERM U1073, Institute for Research and Innovation in Biomedicine, Faculty of Medicine, University of Rouen, Rouen, France

## Abstract

*Background.* Cystic fibrosis-associated liver disease (CFLD) is a major cause of death. The objective of our retrospective study was to describe the relevance of magnetic resonance imaging (MRI) and liver stiffness measurement (LSM) for CFLD evaluation.* Methods.* All cystic fibrosis adult patients evaluated by MRI and LSM were included. MR signs of portal hypertension (PHT), dysmorphia, or cholangitis were collected and LSM expressed in kPa and Metavir.* Results.* Of 25 patients, 52% had abnormal MRI. Median LSM was 5.7 kPa (3.4–9.9). Three patients had F2 score and one had F3 score. In patients with PHT, LSM was 7.85 kPa (3.7–9.9) compared to 5 (3.4–7.5) in others, *p* = 0.02. In patients with abnormal liver function tests, 50% had increased LSM (≥F2), whereas 94% with normal tests had normal LSM (*p* = 0.04). Seven patients had abnormal MRI despite normal ultrasonography.* Conclusions.* MRI and LSM provide useful information on CFLD and may help to screen patients with PHT.

## 1. Background

Cystic fibrosis-associated liver disease (CFLD) leads to important morbidity and mortality [1]. Hepatobiliary disease is the most common nonpulmonary cause of mortality in cystic fibrosis (the third after pulmonary disease and transplant complications). Its prevalence is estimated to involve around one-third of patients regardless of diagnosis methods used (clinical, biochemical, or radiological) and may rise to 72% in autopsy series [2, 3]. This difference is explained by the definition of CFLD. In fact, CFLD definition is controversial. Usually, for diagnosis, two abnormalities, clinical (hepatomegaly, splenomegaly) or biological (elevated liver function tests without other causes, in 3 determinations, during a period of 12 months) or US's signs (liver involvement, portal hypertension, and biliary abnormalities), seem necessary for diagnosis [4].

Dysfunction of the CFTR protein located on the apical pole of the cholangiocyte membranes [5, 6] is responsible for impaired bile transport [7] and focal duct obstruction [8]. Production of proinflammatory chemokines by cholangiocytes, such as Tumor Growth Factor *β*, induces hepatic stellate cell chemotaxis and proliferation. This inflammatory reaction is responsible for primary sclerosing cholangitis-like cholangiopathy, by the production of extracellular matrix [8, 9].

Early recognition is important for diagnosis of fibrosis and prevents its complications, especially portal hypertension (PHT). Tools available for diagnosis of cystic fibrosis-associated liver disease (CFLD) are scarce [10]. Currently annual liver function test (LFT) and ultrasonography (US) are proposed. Colombo et al. proposed CFLD diagnosis criteria, including clinical, biochemical, US, and histological criteria [2]. These criteria have not been validated and have low sensitivity and specificity [11]. Liver biopsy (LB) is an invasive procedure, which is potentially dangerous in CF patients with respiratory weakness and lung distension. Moreover, the focal nature of fibrotic lesions in the initial phases of the disease may lead to inadequate estimation of fibrosis [12]. Lewindon et al. recommended a dual pass LB, to improve the result of CFLD diagnosis to 21% [13]. However, LB is associated with risks, which increase with the number of passes [14]. Furthermore, it is currently difficult to predict which patients will develop cirrhosis with complications. So, LB indications are limited to ruling out severe liver disease in settings of evaluation prior to lung transplantation. Recently, noninvasive tests have been developed to evaluate liver diseases and especially fibrosis [15]. Hepatic and biliary magnetic resonance imaging (MRI) allows extensive exploration of the liver and biliary tract whereas transient elastography is able to estimate fibrosis. This method of fibrosis evaluation has been proved to be reliable in many liver diseases including mainly biliary tract abnormalities such as primary sclerosis cholangitis [16].

The objective of the present cohort study was to describe the relevance of magnetic resonance cholangiography and transient elastography for assessment of cystic fibrosis-associated liver disease in adult patients.

## 2. Methods

### 2.1. Study Subjects and Design

A retrospective one-year cross-sectional cohort study was performed in our cystic fibrosis reference centre at Rouen University Hospital. All adult patients with CF, investigated by hepatobiliary MRI and by transient elastography for liver stiffness measurement (LSM) between July 2009 and July 2010, were included. We excluded patients in whom CFTR-related disorder was limited to one-organ dysfunction (i.e., congenital bilateral absence of vas deferens). Clinical and genetic characteristics were retrospectively collected from patient charts and included meconium ileus, pancreatic insufficiency, diabetes mellitus, body mass index, ΔF508 genotype, alcohol intake (according to the World Health Organisation's definition [17]), ursodeoxycholic acid (UDCA), antibiotics, and antifungal treatments. We also collected biochemical analysis (LFT, platelet counts, prothrombin time, albumin, and renal function) and routine abdominal US results including hepatic dysmorphia or PHT signs. In all patients with abnormal LFT (any test > twice the normal values), additional workup was available including search for hepatitis B, hepatitis C, ferritin, transferrin saturation, and fasting lipid profile. Pulmonary function was collected, including forced expiratory volume.

### 2.2. LSM by Transient Elastography

LSM by transient elastography was measured by Fibroscan® (Echosens®, Paris, France, size M). Ten measurements were taken in 3 different sites in the hepatic right lobe through an intercostal space, with the patient's right arm in maximal abduction. The results are expressed as a mean of 10 valid measurements. To be valid, the measurements had to meet two conditions: (i) an adequate success rate defined as the number of valid measurements divided by the total number of measurements performed greater than or equal to 60% and (ii) an interquartile range (IQR) of less than 30% of the median to take into account the variability of valid measurements. Results were expressed in kilopascal (kPa) and a correspondence table to the Metavir scoring system was used based on previous study of transient elastography in chronic biliary disease [16]. Metavir F0-F1 score corresponded to LSM of ≥7.2 kPa, and F2, F3, and F4 corresponded to ≥7.3 kPa, 9.8 kPa, and 17.3 kPa, respectively [16].

### 2.3. Biliary and Hepatic Magnetic Resonance Imaging

MRI examination was performed with 1.5 Tesla (Philips Achieva, Philips Medical Systems, Best, Netherlands) using a torso phased-array coil. No particular preparation was required. Fasting 4 hours before was required. All patients were placed in supine position with the upper abdomen centred on the coil. The following sequences were performed: (1) T1-weighted sequence, axial image (TR 183 ms, TE 2.3 ms, FOV 70 mm, slice thickness 7 mm, angle 55°, 152 × 432); (2) T2-weighted sequence, axial SPAIR (TR 4459 ms, TE 70 ms, FOV 76 mm, slice thickness 6 mm, angle 90°, 218 × 320); (3) T2-weighted sequence, axial HR (TR 1573 ms, TE 100 ms, FOV 79 mm, slice thickness 7 mm, angle 90°, 341 × 560); (4) T2-weighted sequence diffusion 2b (TR 1489 ms, TE 59 ms, FOV 90 mm, slice thickness 6 mm, 92 × 67); (5) 3D MR cholangiogram (TR 1341 ms, TE 574 ms, FOV 100 mm, slice thickness 2.4 mm, angle 90°, 221 × 560); (6) in and out phase sequence (TR 175 ms, TE 2.3 ms (in), 4.8 ms (out), FOV 40°, slice thickness 4 mm, angle 80°, 224 × 192). Abdominal radiologists (CSC and EK) reviewed all MRI results blinded to clinical or biochemical parameters and reached decisions by consensus. Native and 3D MIP (maximum intensity projection) reconstructions were analyzed on a workstation (EasyVision, Philips Medical Systems, Best, Netherlands). The following items were studied for each patient using a standardized scale: atrophy of either right or left hepatic lobe and/or hypertrophy of the caudate lobe, marked lobulations of liver surface, first-segment hypertrophy, splenomegaly (long axis superior to 12 cm), portal vein dilatation (diameter superior to 12 mm), splenic vein dilatation, intrahepatic or extrahepatic biliary duct irregularity (segmental strictures and dilatations), ascites, and steatosis. Pancreatic patterns were also noted: partial or total pancreatic fatty involution, Wirsung duct irregularity, and pancreatic cysts.

PHT was diagnosed in the presence of one or more of these signs: collateral circulation, portal vein dilatation, splenic vein dilatation, or splenomegaly. Hepatic dysmorphia was diagnosed in the presence of one or more of these signs: atrophy of either right or left hepatic lobe, hypertrophy of the caudate lobe, marked lobulations of liver surface, and first-segment hypertrophy.

We studied the results of LSM and hepatic MRI, according to the results of LFT or US.

### 2.4. Statistical Analysis

Statistical analysis was conducted using SAS software version 9.3 (SAS Institute, Cary, NC, USA). SI units were used for all laboratory values with data summarized using mean ± standard deviation (SD) for continuous variables and number (%) for all recorded categorical variables describing the study population.

LSM are expressed in kPa as median (IQR). Student's* t*-test was used to compare continuous variables between CF patients included and the whole CF cohort as appropriate, whereas categorical variables were compared using chi-square test.

Regarding concordance between study methods, agreement was evaluated by Kappa-test: 1 = perfect agreement, 1–0.8 = almost perfect agreement, 0.8–0.6 = good agreement, 0.6–0.2 = moderate and fair agreement, and less than 0.20 = slight agreement. To assess the diagnostic performance of LSM for prediction of PHT, the area under the receiver operating curve (AUROC) was calculated. Optimal LSM for prediction of PHT was identified by estimating sensitivity and specificity for various cut-offs.

Prevalence of abnormalities in MRI and LSM was compared regarding the presence or not of LFT and/or US abnormalities using chi-square test and Fisher's exact test.

All statistical significance was taken at 95% confidence interval and *p* value of less than 0.05 was considered as significant.

## 3. Results

### 3.1. Patients' Characteristics

**Figure 1:**
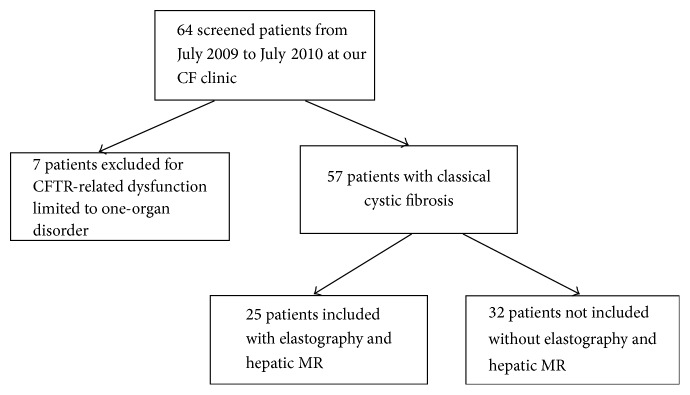
Flow chart for inclusion in our study.

Of 64 adult CF patients followed up at our tertiary care center, 25 were included (Figure 1). Demographic characteristics at baseline are summarized in Table 1. Characteristics of studied patients were not statistically different compared to our whole CF population. However, patients included were more likely to receive UDCA (40% versus 13%, *p* = 0.028) and antifungal drugs (76% versus 31%, *p* < 0.001) than nonincluded patients. Only 6 patients included in our study had abnormal LFT and/or US abnormalities (hepatic dysmorphia, PHT signs, and gallstones). FEV1 median is 67.6% (50.4–84.8) in our 25 patients versus 71.2% (47.5–94.9) in the whole CF cohort (*p* = 0.33).

### 3.2. MRI Results (Table 2)

**Figure 2:**
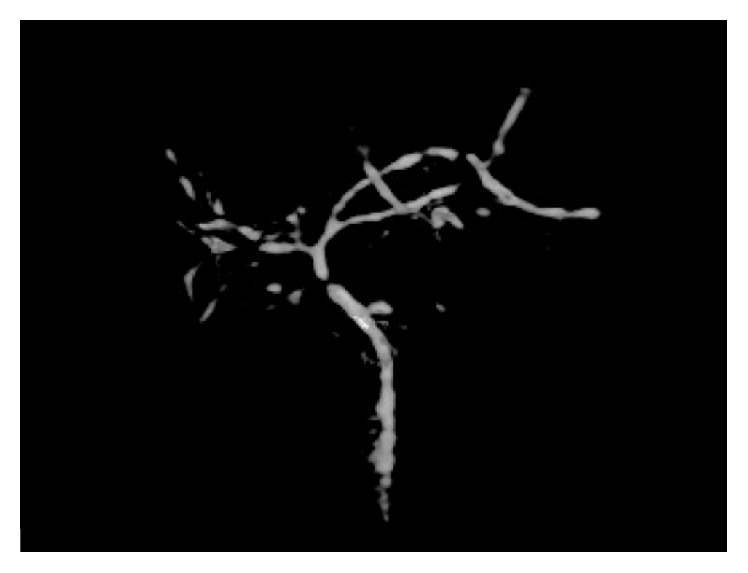
3D MRI cholangiogram shows intrahepatic and extrahepatic biliary duct irregularity, with choledochus stenosis.

At least one abnormal MRI sign was found in 13 patients (52%). We evidenced isolated hepatic dysmorphia, PHT, and isolated biliary tract abnormality in 3 patients (12%), 5 patients (20%), and 1 patient (4%), respectively (Figure 2). Two patients combined hepatic dysmorphia, PHT signs, and biliary abnormalities (8%). We found no liver steatosis or ascites in our series. Other MRI abnormalities found were pancreatic cysts in 10 patients (40%) and Wirsung duct irregularity in 5 patients (20%). Partial and total pancreatic fatty involution were found in 5 patients (20%) and 19 patients (76%), respectively. Two patients had gallstones without complications.

### 3.3. LSM Results

Of the 25 patients included, 2 were excluded due to success rate of less than 60% in transient elastography, with mismatch between sites of measurement. Taking into account the median of the 10 highest measures for each patient, the median value of LSM was 5.7 kPa (3.4–9.9) in this cohort. Fibrosis scores were as follows: F0-F1 in 19 patients (82.6%), F2 in 3 patients (13%), and F3 in 1 patient (4.3%), and no patient reached the F4 stiffness threshold. All patients with a score greater than or equal to F2 were men (*p* = 0.019). In patients with evidence of hepatic disease obtained by any other means, mean LSM was 6 kPa (3.4–9.9), versus 4.9 kPa (3.9–7.3) in patients without CFLD (*p* = 0.3).

**Figure 3:**
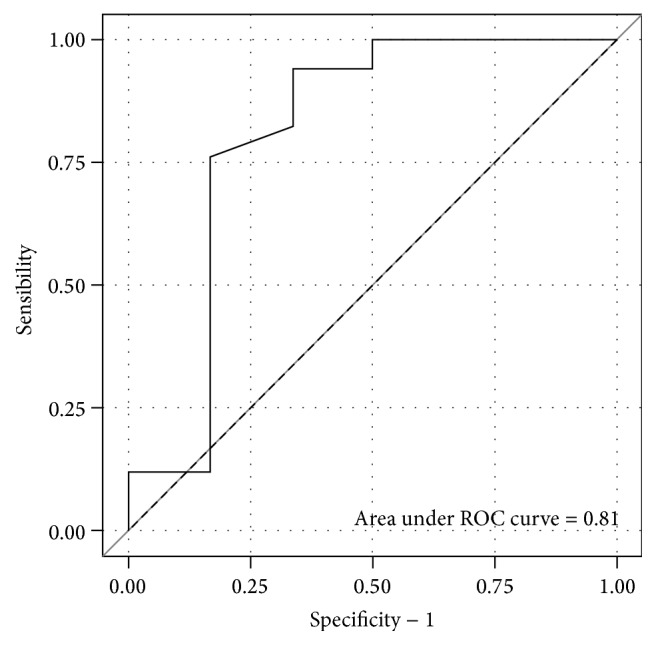
ROC curve for liver stiffness measurement predicting the presence of portal hypertension.

In patients with evidence of PHT, mean LSM was 7.85 KPa (3.7–9.9) compared to 5 kPa (3.4–7.5) in patients with no evidence of PHT (*p* = 0.02). The AUROC for LSM predicting the presence of PHT was 0.80 [95% CI = [0.53 ; 1.0]] (Figure 3). LSM cut-off of 6.3 kPa predicted the presence of PHT with 66.7% sensitivity, 94.1 specificity, 80.0 positive predictive value, and 88.9 negative predictive value. No patient with PHT on imaging technique had bleeding prior to or during the study. Endoscopic evaluation was available for 2 patients with PHT: one was normal, and the second showed PHT gastropathy.

### 3.4. MRI and LSM Concordance

For diagnosis of CFLD, there was low concordance between transient elastography and MRI (*k* = 0.25). Kappa values observed were 0.5 for PHT assessment with agreement reaching the threshold of “moderate” and 0.24 for fibrosis.

### 3.5. Magnetic Resonance Cholangiography and Liver Elastography Results according to Routine Testing Status

With a median of 2 LFT per patient, only 6 patients had at least 1 abnormal LFT (28%) during the study period.

Of the 60% of patients with routine US, 5 had abnormal US findings: 3 patients with signs of PHT and 2 with gallstones without evidence of any complications. All patients with abnormal US findings had at least 1 abnormal LFT.

FEV1 was not different between patients with or without PHT (*p* = 0.59).

Table 3 summarizes LSM and MRI findings according to routine status testing (LFT and US). Of the 6 patients with abnormal LFT and US, 50% had significant increase in liver stiffness (≥F2), whereas 94% of the 17 patients with normal routine status testing had normal LSM, *p* = 0.04.

## 4. Discussion

Noninvasive assessment of CFLD is an emerging topic with important implications [15]. The present study reports a combined approach using MRI and transient elastography for liver stiffness evaluation in adults with CF. We found overall prevalence of 52% for MRI abnormalities including 50% of patients with previous normal ultrasonography. Liver stiffness was high only in a few individuals (1 F3 and 3 F2), but we found an association between higher value of LSM and PHT pointing to the potential role of LSM in PHT screening.

The prevalence of liver disease varies considerably across studies, depending on the diagnostic methods used, from 27 to 35% [2, 3]. Our observed prevalence seems higher. It may be explained first by a selection bias in our cohort with only adult patients. Prevalence depends on definition too. For some people, definition may include cirrhosis or PHT, which decreased prevalence to 7% [2]. Secondly, the rate of prescription of UDCA was slightly higher in patients studied (see Table 1) than in nonincluded patients and also by the imaging technique used to search for lesions. MRI is a technique with high sensitivity and specificity in evaluation of the biliary tract [18]. MRI-based imaging studies in CF are scarce. Durieu et al. showed that, in patients with abnormal liver function tests, clinical abnormalities, and abnormal US, all presented abnormalities on MRI. When all previous explorations were normal, half still had abnormalities on MRI, including cholangiopathy [19]. This confirms the importance of evaluation and MRI in evaluating other techniques, for diagnosis of CFLD. MRI has now become a widely available technique in tertiary reference centers. Moreover, since it is a not radiating method it may be repeated with very few contraindications. We decided not to use contrast agents in these fragile patients to minimize the invasiveness of the examination. In addition, the sequences used for the explorations in question do not necessarily require contrast agents. MRI application in practice seems easy. MRI is also able to diagnose pancreatic abnormalities and we observed pancreatic lesions in the majority of patients. This additional information may contribute to enhancing the impact of MRI on decision-making processes for these patients. Without MRI, 6 out of 25 patients in our series would not have been diagnosed by ultrasonography. This observation further emphasizes the difficulty of diagnosing CFLD. In our study, all patients had steatosis criteria, which is unusual compared to other studies [20]. However, many techniques can help in the diagnosis of steatosis: LB, US, and MRI. These techniques have different efficiencies. So we can explain our low prevalence, by the technique used in this study.

Transient elastography for LSM is a promising tool in the field of hepatology. It has replaced LB in several chronic liver diseases including viral hepatitis [15]. Although it is used in current practice, LSM remains to be validated for all cholestatic diseases. As a consequence, there is no correspondence between measures expressed in kPa and the Metavir scoring system which is proposed for CFLD. Nevertheless, in the present report, we have used thresholds developed in chronic cholestatic diseases considering the pathophysiology to be close [16]. This scoring system has guided clinical practices in order to adapt surveillance and therapeutic options. In addition to being an invasive and potentially dangerous procedure in the presence of respiratory weakness and lung distension, LB has limited reliability, due to the focal distribution of lesions. This often results in under- or overestimation of disease and has led authors to propose dual biopsies in pretransplantation workup.

Transient elastography however obviates this risk and multiple measurements may be appropriate in overcoming underestimation. We chose to apply the “rule of 10 valid measurements” on three sites but it may be worth considering other approaches including increased number of sites of measurement in such clinical conditions.

As others, we found median values of LSM to be quite low. Witters et al. reported a mean of 5.6 kPa in pediatric patients [21] and Kitson et al. reported a mean of 6.55 kPa in 50 unselected adults with CF [22]. In patients with established CFLD, mean LSM reached, respectively, 11.2 in the pediatric cohort [21] and 8.1 kPa in the adult cohort [22]. For cirrhosis, Karlas et al. found a mean of 7,95 kPa [23]. In our study, the mean was 5.7 in all the cohort and was 7.8 kPa for patients with demonstrated liver abnormalities.

It is usual to consider that LSM is a valuable tool to distinguish low fibrosis (F0–F2) and F3-F4. LSM may be a valid approach to screen early CFLD. To do so, cut-off values for CFLD are mandatory. For the first time, Robertson et al. reported a cut-off at 8.83 kPa [20]. Later, Kitson et al. found a cut-off at 6.8 kPa [22]. In our series, we limited assessment of LSM value to its performance in predicting portal hypertension for which no diagnosis controversies exist. We found a cut-off at 6.3 kPa. This choice was based on the absence of consensus definition for CFLD outside the field of LB results.

Our results show a link between increased LSM and PHT. This relationship is present even in patients without evidence of cirrhosis. We ruled out cirrhosis in the absence of F4 stage in LSM and in 75% of patients in the absence of dysmorphic liver feature on MRI. This suggests that noncirrhotic portal hypertension (NCPH) is secondary to other mechanisms. These mechanisms have already been described in primary biliary cirrhosis [24] and primary sclerosing cholangitis [25, 26]. A presinusoidal block has been described in the early stage of primary biliary cirrhosis followed by a sinusoidal block in later fibrosis stages [24]. The main hypothesis is that NCPH is secondary to a vascular component. In NCPH, histopathology reveals nodular regenerative hyperplasia (NRH) and/or obliterative portal venopathy (OPV), responsible for an increase in intrahepatic resistance [26, 27]. In cases of NRH, patients frequently had splenomegaly [28]. OPV can be explained by biliary inflammation-related obliteration of portal veins [26, 29]. We can speculate that this natural history may be present in CF. In CF, platelets are hyperactive and are responsible for microthrombosis [30]. Early endothelial dysfunction related to CF vasculitis has been described [31]. All of these abnormalities could contribute to OPV. In fact, Witters et al. describe 7 cases of NCPH in CF patients, with presinusoidal block pressure gradient. In biopsy, they found portal branch venopathy [32].

Early treatment of CFLD is of increasing importance in the management of patients with cystic fibrosis and prevention of severe liver damage. CFLD standard treatment is based on UDCA at a dose of 20–30 mg/kg/day [33]. Immediate treatment upon diagnosis is currently the only proven and effective therapy [34, 35]. Some studies objectify improved liver function tests and histological damage by UDCA in patients with CFLD. Nevertheless, despite such improvements, there is a lack of evidence for changes in prognosis [33]. An increase in the life expectancy of patients with CF is linked to increased prevalence of CFLD. It is important to optimize disease management prior to lung transplantation or even consider double lung-liver transplant. Currently, none of our patients had transplantation.

## 5. Conclusion

Although it is a retrospective design on a small sample size, our study, based on noninvasive liver explorations using MRI and LSM, provides useful and additional information on assessment of CFLD. The results of such investigations are likely to impact clinical practice justifying further studies. Transient elastography for assessment of liver stiffness may soon play a central role in the noninvasive technique of portal hypertension screening. MRI seems essential for the CFLD diagnosis.

## Figures and Tables

**Table 1:** Patients' characteristics at study enrolment.

Patients' characteristics	Studied patients *n* = 25 (%)	Whole CF cohort *n* = 57 (%)	*p*
Gender (male/female)	0.46	0.50	1
Median age (yrs [min.–max.])	25 [18–43]	30.1 [18–62]	0.12
Meconium ileus	4 (16%)	12 (21%)	0.76
Pancreatic insufficiency	22 (88%)	49 (85.9%)	1
Genotype			
ΔF508-ΔF508	11 (44%)	20 (35.1%)	0.46
ΔF508, other mutations	12 (48%)	31 (54%)	0.63
2 other mutations	1 (4%)	6 (10%)	1
Medium BMI (kg/m^2^ [min.–max.])	19.3 [17.0–29.4]	21.4 [17.0–32.9]	0.11
Alcohol	0 (0%)	0 (0%)	1
Diabetes mellitus	4 (16%)	13 (22.8%)	0.57
Dyslipidemia	1 (4%)	1 (1.7%)	0.52
Antibiotics	17 (68%)	41 (72%)	0.79
Antifungal drugs	19 (76%)	29 (51%)	0.051
UDCA treatment	10 (40%)	14 (24%)	0.19
Creatinine clearance ≥ 70 mL/min	25 (100%)	57 (100%)	1

FEV1 (%)	67.6% [50.4–84.8]	71.2% [47.5–94.9]	0.33

BMI: body mass index; UDCA: ursodeoxycholic acid.

**Table 2:** MRI results.

Diagnosis in MRI	Specific abnormalities in MRI	*N*	Total number (%)
Abnormal MRI			13 (52%)

Hepatic dysmorphia	Included lobulation of liver surface	3	3 (12%)
	Included first-segment hypertrophy	0	

Portal hypertension	Included splenomegaly	5	5 (20%)
	Included portal vein dilatation	2	
	Included splenic vein dilatation	1	

Isolated biliary abnormalities	Intrahepatic	1	1 (4%)
	Extrahepatic	0	

Hepatic dysmorphia and portal hypertension		0	0 (0%)

Hepatic dysmorphia and biliary abnormalities	Included lobulation of liver surface and intrahepatic abnormalities	1	1 (4%)

Portal hypertension and biliary abnormalities	Included portal vein hypertrophy and intrahepatic abnormalities	1	1 (4%)

Hepatic dysmorphia, portal hypertension, and biliary abnormalities	Included splenomegaly, lobulation of liver surface, first-segment hypertrophy, and intrahepatic and extrahepatic abnormalities	2	2 (8%)

Normal MRI			12 (48%)

Total			25 (100%)

*N*: number of patients; MRI: magnetic resonance imaging.

**Table 3:** Results of LSM and MRI according to the results of liver function tests and abdominal ultrasonography.

LFT or US abnormalities		*p*
Yes (*n* = 6)	No (*n* = 17)	
50% F0-F1 (*n* = 3)	94% F0-F1 (*n* = 16)	0.04
50% ≥ F2 (*n* = 3)	6% ≥ F2 (*n* = 1)	

33.3% normal MRI (*n* = 2)	58.8% normal MRI (*n* = 10)	0.37
66.7% abnormal MRI (*n* = 4)	41.2% abnormal MRI (*n* = 7)	

MRI: magnetic resonance imaging; US: ultrasonography; LFT: liver function test.

## Abbreviations

CF: Cystic fibrosis
CFLD: Cystic fibrosis-associated liver disease
LB: Liver biopsy
LSM: Liver stiffness measurement
LFT: Liver function test
MRI: Magnetic resonance imaging
kPa: Kilopascal
PHT: Portal hypertension
CI: Confidence interval
UDCA: Ursodeoxycholic acid.
